# miR-320a functions as a suppressor for gliomas by targeting SND1 and β-catenin, and predicts the prognosis of patients

**DOI:** 10.18632/oncotarget.14975

**Published:** 2017-02-01

**Authors:** Huining Li, Lin Yu, Jing Liu, Xiuwu Bian, Cuijuan Shi, Cuiyun Sun, Xuexia Zhou, Yanjun Wen, Dan Hua, Shujun Zhao, Linlin Ren, Tongling An, Wenjun Luo, Qian Wang, Shizhu Yu

**Affiliations:** ^1^ Department of Neuropathology, Tianjin Neurological Institute, Tianjin Medical University General Hospital, Tianjin, China; ^2^ Tianjin Key Laboratory of Injuries, Variations and Regeneration of the Nervous System, Tianjin, China; ^3^ Key Laboratory of Post-trauma Neuro-repair and Regeneration in Central Nervous System, Ministry of Education, Tianjin, China; ^4^ Department of Biochemistry and Molecular Biology, School of Basic Medical Sciences of Tianjin Medical University, Tianjin, China; ^5^ Institute of Pathology and Southwest Cancer Center, Southwest Hospital, Third Military Medical University, Chongqing, China; ^6^ Laboratory of Hormone and Development, Ministry of Health, Institute of Endocrinology, Tianjin Medical University, Tianjin, China

**Keywords:** gliomas, miR-320a, SND1, β-catenin, prognosis

## Abstract

miR-320a downexpression contributes to tumorigenesis in several human cancers. However, the relevance of miR-320a to prognosis, proliferation and invasion in gliomas remains unclear. In this study, we demonstrated that miR-320a expression was decreased in human glioma tissues and cell lines. Moreover, miR-320a expression was inversely correlated with glioma grades and Ki-67 index, but positively correlated with patients’ survival. Contrarily, SND1 and β-catenin expressions were positively correlated with glioma grades and Ki-67 index, but inversely correlated with miR-320a expression and patients’ survival. Furthermore, two subgroups with distinct prognoses in our glioma patients of different grade, IDH status, age and KPS were identified according to expression of miR-320a, SND1 or β-catenin. Cox regression showed that miR-320a and SND1 were independent predictors and β-catenin was an auxiliary predictor for patients’ survival. miR-320a overexpression suppressed the G1/S phase transition, proliferation, migration and invasion of glioblastoma cells. Mechanistically, we validated SND1 and β-catenin as direct targets of miR-320a, and found that miR-320a overexpression increased SND1-inhibited tumor suppressor p21^WAF1^ and decreased Smad2, Smad4, MMP2, MMP7 and cyclinD1, the pivotal downstream effectors of SND1 or β-catenin. Our findings demonstrate the potential values of miR-320a, SND1 and β-catenin as prognostic biomarkers and therapeutic candidates for malignant gliomas.

## INTRODUCTION

Gliomas are the most frequent primary brain tumors [1, 2]. High-grade gliomas, such as glioblastoma multiform (GBM), are characterized by rapid growth and relentless invasion, which makes radical resection almost impossible for them [3]. Despite the progress in radiotherapy and chemotherapy, the prognosis of GBM patients remains dismal, with a median survival time of 14.6 months [4]. Therefore, the new approaches of diagnosis, prognostic evaluation and therapy used for gliomas have to be explored by understanding their genetic and epigenetic changes.

Human miR-320 family has 5 mature members termed as miR-320a to e. They are encoded by the loci on chromosome 1, 8, 13, 19 and X, respectively. The aberrant expression of miR-320a has been reported in various malignant tumors [5–7]. In bladder and colon cancers, its expression level is extremely low [8–9], whereas in retinoblastoma, higher miR-320a level is associated with a more malignant phenotype [10], suggesting that function of this miRNA may be distinct and even opposite in different tumors. However, the expression pattern, prognostic significance and biologic functions of miR-320a remain to be fully elucidated in gliomas.

Staphylococcal nuclease domain-containing 1 (SND1), also known as Tudor-SN or p100 coactivator, and β-catenin are overexpressed in various malignant tumors including malignant gliomas [11–16]. However, the upstream mechanisms inducing SND1 and β-catenin overexpressions in gliomas remain poorly understood. In this paper, we report for the first time that miR-320a inhibits the proliferation, migration and invasion of glioma cells by directly silencing SND1 and β-catenin, and identify miR-320a and SND1 as independent predictors and β-catenin as an auxiliary predictor for the survival of glioma patients. Our findings also indicate that miR-320a downexpression is an important cause leading to SND1 and β-catenin overexpressions, and suggest that miR-320a, SND1 and β-catenin are potential therapeutic candidates for malignant gliomas.

## RESULTS

### miR-320a is decreased in gliomas and its higher expression predicts better prognosis

**Figure 1:**
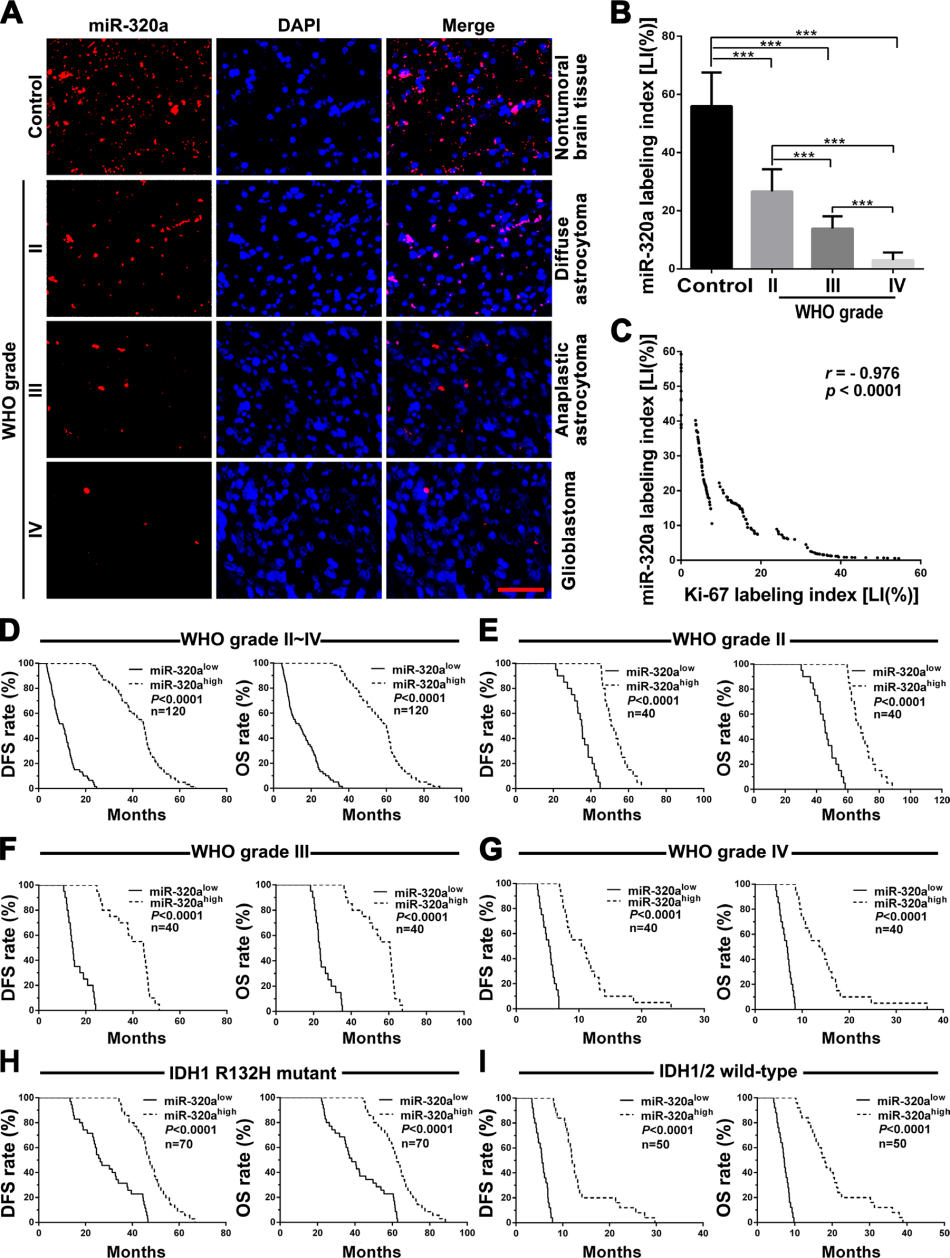
miR-320a expression correlates with grades, proliferation, IDH status and prognosis in human gliomas **A**. Representative images of miR-320a ISH with LNA-modified probe. Scale bar, 50μm. **B**. Comparisons among groups of miR-320a expression level [Labeling index (%), LI] in the FFPE samples of 120 gliomas and 20 nontumoral control brain tissues. **C**. Pearson correlation analysis between miR-320a and Ki-67 expressions in our FFPE samples. The miR-320a LI and Ki-67 LI of each sample were calculated with Leica Image Pro Plus 5.0 software according to the percentage ratio of positive cell number to total cell number and the data in (B) are presented as the mean ± SD. *** *P*<0.001. **D-I**. Kaplan-Meier analysis of the correlation between miR-320a and DFS (left) or OS (right) of all the glioma patients (D) and the patients with WHO grade II (E), grade III (F), grade IV (G), IDH1 R132H mutant (H) and IDH1/2 wild-type (I) gliomas. Patients were stratified into high and low expression subgroups using the median of miR-320a LIs.

To identify relationships between miR-320a expression in gliomas and histopathological grades, cell proliferation or patients’ prognoses in the same grade, IDH status, age and KPS groups, ISH and IHC were applied to detect endogenous miR-320a and Ki-67 expressions in the FFPE specimens of 120 gliomas and 20 nontumoral brain tissues from human. We demonstrated that miR-320a expression in gliomas was lower than that in the control (*P*<0.001) and that its expression was significantly decreased with the elevation of glioma grades and was the lowest in GBM (*P*<0.001; Figure 1A and 1B). Moreover, miR-320a expression was inversely correlated with proliferation index (Ki-67 LI; *r=*-0.976, *P*<0.0001; Figure 1C and Supplementary Figure 1A and 1B). Stem-loop qRT-PCR detection further verified that miR-320a expression in 7 human GBM cell lines was also significantly reduced in comparison with human astrocyte cell line UC2 (Supplementary Figure 2). Kaplan-Meier analysis showed that the patients with higher level of miR-320a had longer disease-free survival (DFS; *P*<0.0001) and overall survival (OS; *P*<0.0001; Figure 1D). Significantly, we found that glioma patients in the same grade, IDH status, age and KPS groups could be divided into two subgroups with different outcomes based on miR-320a expression, i.e., the higher expression of miR-320a was, the better prognosis of patients (DFS: *P*<0.0001; OS: *P*<0.0001; Figure 1E-1I and Supplementary Figure 3A-3D). The prognostic value of miR-320a in GBM was further verified in GBM patients from TCGA database (DFS: *P* = 0.0036; OS: *P* = 0.0317; Supplementary Figure 4). Both the multivariate and univariate analyses showed that miR-320a was an independent predictor for DFS and OS of glioma patients (Table 1 and Supplementary Table 1). These data indicate the inverse association of miR-320a expression with the grades and cell proliferation of gliomas, and reveal that miR-320a is a potential prognostic biomarker for glioma patients.

**Table 1:** Multivariate analysis for DFS and OS in patients with gliomas

Factors	DFS		OS	
	HR(95%CI)	*P*	HR(95%CI)	*P*
Gender	1.238(0.748-2.050)	0.407	1.324(0.822-2.134)	0.249
Age	1.000(0.982-1.019)	0.994	0.990(0.973-1.007)	0.254
Predominant side	0.769(0.499-1.186)	0.234	0.618(0.411-0.931)	0.021
Predominant location	0.830(0.580-1.187)	0.307	0.922(0.666-1.275)	0.623
KPS	0.969(0.936-1.002)	0.068	0.982(0.952-1.013)	0.253
IDH status	0.000(0.000-0.007)	0.001	0.000(0.000-0.021)	0.001
miR-320a LI	0.635(0.536-0.751)	<0.0001	0.707(0.616-0.811)	<0.0001
SND1 LI	1.655(1.219-2.247)	0.001	1.697(1.202-2.395)	0.003
β-catenin LI	1.326(0.928-1.894)	0.122	1.015(0.730-1.412)	0.928
Ki-67 LI	2.924(1.267-6.744)	0.012	2.428(1.195-4.932)	0.014

Abbreviations: HR, hazard ratio; CI, confidence interval; KPS, Karnofsky performance score; LI, labeling index.

### miR-320a suppresses the proliferation, migration and invasion of GBM cells

**Figure 2:**
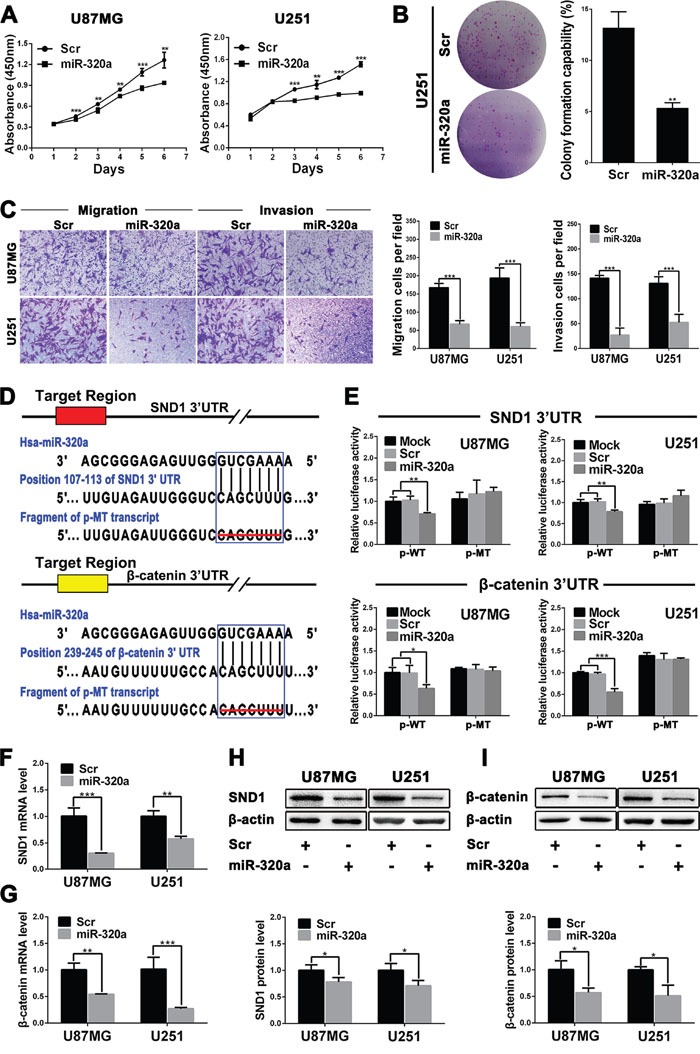
miR-320a functions as a glioma suppressor by directly targeting SND1 and β-catenin **A**. Growth curves from U87MG and U251 cells transfected with scrambled control sequence (Scr) or miR-320a mimics (miR-320a) assessed by CCK8 assay. **B**. Colony formation efficiencies of the transfected U251 cells. **C**. Representative images (left) and numbers (right) of the indicated migratory and invasive cells analyzed by transwell assay. **D**. Predicted miR-320a binding sites in the 3′-UTRs of SND1 and β-catenin by TargetScan and the designed mutant 3′-UTRs in which miR-320a binding sites were deleted. **E**. Dual luciferase reporter assays in U87MG and U251 cells transfected with p-WT or p-MT alone (Mock) and cotransfected with p-WT or p-MT and Scr or miR-320a; p-WT=the reporter plasmid of wild-type SND1 or β-catenin 3′-UTR, p-MT=the reporter plasmid of mutant-type SND1 or β-catenin 3′-UTR. The data were normalized according to the ratio of firefly luciferase activity to renilla luciferase activity. **F** and **G**. SND1 and β-catenin mRNA levels in the indicated cells analyzed by qRT-PCR and normalized against GAPDH. The ratios of SND1/GAPDH and β-catenin/GAPDH in the Scr-transfected cells were arbitrarily set to 1.0. **H** and **I**. SND1 and β-catenin protein levels in the indicated cells detected by Western blot and normalized against β-actin. All the experiments were performed at least in triplicate and the data are presented as the mean ± SD. * *P*<0.05, ** *P*<0.01, *** *P*<0.001.

Prompted by the above findings, we examined the tumor suppressive effects of miR-320a on GBM cell lines by transient mimics transfection. CCK8 assays showed that miR-320a could effectively inhibit the proliferation of U87MG and U251 cells compared with Scr control 48 or 72 h after transfection (*P*<0.01~0.001; Figure 2A). Moreover, miR-320a-transfected U251 cells displayed lower colony formation efficiency (*P*<0.01; Figure 2B), further confirming its long-term anti-proliferative effect. Furthermore, miR-320a transfection dramatically suppressed the migration and invasion of U87MG and U251 cells, as gauged by the transwell assays (*P*<0.001; Figure 2C) and wound healing assays (*P*<0.05~0.001; Supplementary Figure 5). The results indicate that miR-320a is an important inhibitor of the proliferation, migration and invasion of GBM cells.

### SND1 and β-catenin are the direct targets of miR-320a in GBM cells

We ascertained 47 mRNAs co-expressing with miR-320a using the data from TCGA database, but only expressions of the six mRNAs had highly negative correlation with miR-320a expression in GBM (Supplementary Table 2). We focused on SND1 and β-catenin because they were important glioma promoters. TargetScan and miRanda predictions revealed that both the 3′-UTRs of SND1 and β-catenin mRNAs contained a conserved miR-320a binding site (Figure 2D). The dual-luciferase assay showed that miR-320a could effectively suppress the luciferase activity delivered by recombinant reporter vectors with wild type 3′-UTRs of SND1 and β-catenin in U87MG and U251 cells (*P*<0.05~0.001), whereas the mutant 3′-UTRs without miR-320a binding sites failed to exert the same effect (Figure 2E). Moreover, the qRT-PCR and Western blot detections further confirmed that miR-320a transfection significantly decreased the mRNAs and proteins of SND1 and β-catenin in U87MG and U251 cells (*P*<0.05~0.001; Figure 2F-2I). The results demonstrate that miR-320a directly binds with SND1 and β-catenin 3′-UTRs, and inhibits their protein expressions through inducing the mRNA degradation in GBM cells.

### SND1 and β-catenin overexpressions are associated with miR-320a downexpression and poorer prognosis in human gliomas

**Figure 3:**
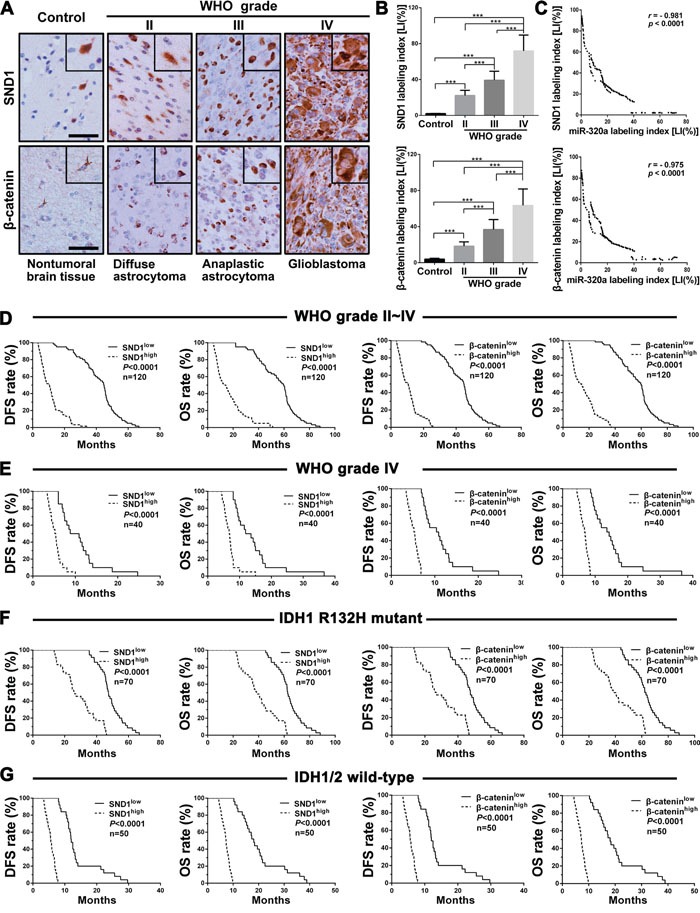
SND1 and β-catenin expressions correlate with grades, miR-320a expression, IDH status and prognosis in human gliomas **A**. Representative images of SND1 and β-catenin IHC detection. Scale bar, 50 μm. **B**. Comparisons among groups of SND1 and β-catenin expression levels [Labeling index (%), LI] in the FFPE samples of 120 gliomas and 20 nontumoral control brain tissues. The computing method of SND1 LI and β-catenin LI was the same as that of miR-320a (see Figure 1 legend) and the data in (B) are presented as the mean ± SD. *** *P*<0.001. **C**. Pearson correlation analysis between the expressions of miR-320a and SND1 or β-catenin in our FFPE samples. **D-G**. Kaplan-Meier analysis of the correlation between SND1 or β-catenin and DFS (left) or OS (right) of all the glioma patients (D) and the patients with grade IV (E), IDH1 R132H mutant (F) and IDH1/2 wild (G) gliomas. Patients were stratified into high and low expression subgroups using the median of SND1 or β-catenin LIs.

We then detected SND1 and β-catenin in the above FFPE specimens of gliomas and brain tissues by IHC, and found that SND1 and β-catenin expressions were higher in gliomas than in brain tissues and were significantly increased with the elevation of glioma grades (*P*<0.001; Figure 3A and 3B). Furthermore, SND1 and β-catenin LIs in gliomas were negatively correlated with miR-320a LI (*r* = −0.981 or −0.975, *P*<0.0001; Figure 3C) and positively correlated with Ki-67 LI (*r*=0.984 or 0.975, *P*<0.0001; Supplementary Figure 1C and 1D). Kaplan-Meier analyses demonstrated that the high levels of SND1 and β-catenin predicted a short-term DFS (*P*<0.0001) and OS (*P*<0.0001; Figure 3D) in glioma patients. Moreover, the glioma patients in the same grade, IDH status, age and KPS groups could also be divided into two subgroups with different outcomes based on SND1 and β-catenin expressions, i.e., the higher their expressions were, the poorer prognosis of patients (DFS: *P*<0.0001; OS: *P*<0.0001; Figure 3E-3G, Supplementary Figure 6A and 6B, and Supplementary Figure 7A-7H). Multivariate and univariate analyses ascertained SND1 as an independent predictor and β-catenin as an auxiliary predictor for DFS and OS of glioma patients (Table 1 and Supplementary Table 1). These data identify the positive correlations of SND1 and β-catenin expressions with the grades and cell proliferation of gliomas, reveal that they are potential prognostic biomarkers for glioma patients, and indicate that miR-320a downexpression is an important cause inducing SND1 and β-catenin overexpressions in gliomas.

### miR-320a suppresses GBM cell proliferation by targeting SND1 and β-catenin

**Figure 4:**
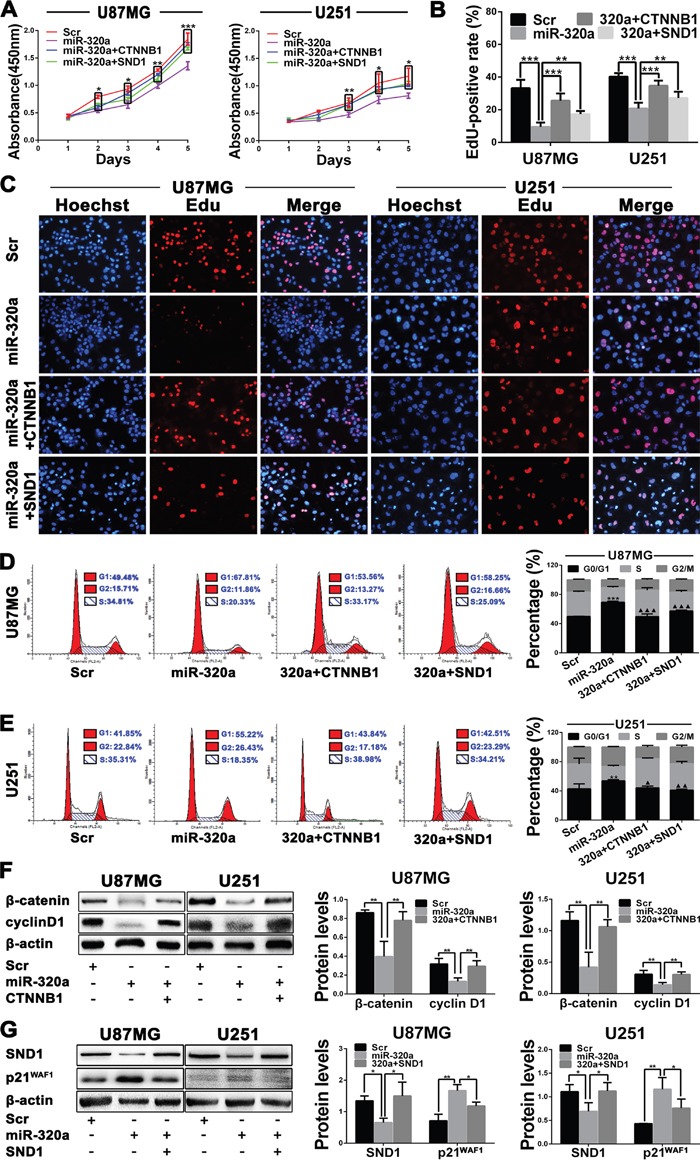
miR-320a suppresses GBM cell proliferation by targeting β-catenin and SND1 **A**. Growth curves from U87MG and U251 cells transfected with Scr or miR-320a and cotransfected with miR-320a plus plasmid expressing β-catenin (miR-320a+CTNNB1) or SND1 (miR-320a+SND1) assessed by CCK8 assay. **B and C**. EdU-positive rate (B) and representative images (C) in the indicated cells assessed by EdU assay. **D and E**. Representative images (left) and percentage of each phase cells (right) in the indicated cells assessed by FCM. **F and G**. Western blot analyses of β-catenin, cyclin D1, SND1 and p21^WAF1^ (left), and comparisons among groups of their expressions (right) in the cells as indicated. Their relative expression levels were normalized against β-actin. All the experiments were performed at least in triplicate and the data are presented as the mean ± SD. * *P*<0.05, **P<0.01, ***/^▲▲▲^ *P*<0.001. Compared with Scr group* and with miR-320a group^▲^ in FCM data.

To investigate the underlying mechanisms by which miR-320a suppresses glioma cell proliferation, U87MG and U251 cells were alone transfected with Scr or miR-320a mimics and co-transfected with miR-320a mimics plus the plasmid expressing β-catenin (miR-320a+ CTNNB1) or SND1 (miR-320a+SND1). The analyses of CCK8, EdU and flow cytometry confirmed that miR-320a transfection significantly restrained the proliferation of GBM cells by inducing their G1 phase arrest, whereas β-catenin and SND1 overexpressions could abrogate the inhibition of miR-320a on the proliferation and cell cycle of GBM cells (*P*<0.05~0.001; Figure 4A-4E). Furthermore, miR-320a transfection reduced cyclin D1 and increased p21^WAF1^ (*P*<0.01) by decreasing β-catenin or SND1 (*P*<0.05~0.01) in GBM cells, which could also be reversed by β-catenin or SND1 overexpression (*P*<0.05~0.01; Figure 4F and 4G). These findings reveal that miR-320a decreases cyclin D1 and increases p21^WAF1^ by directly silencing β-catenin or SND1, thereby suppressing the G1/S phase transition and proliferation of GBM cells.

### miR-320a inhibits GBM cell migration and invasion by targeting SND1 and β-catenin

**Figure 5:**
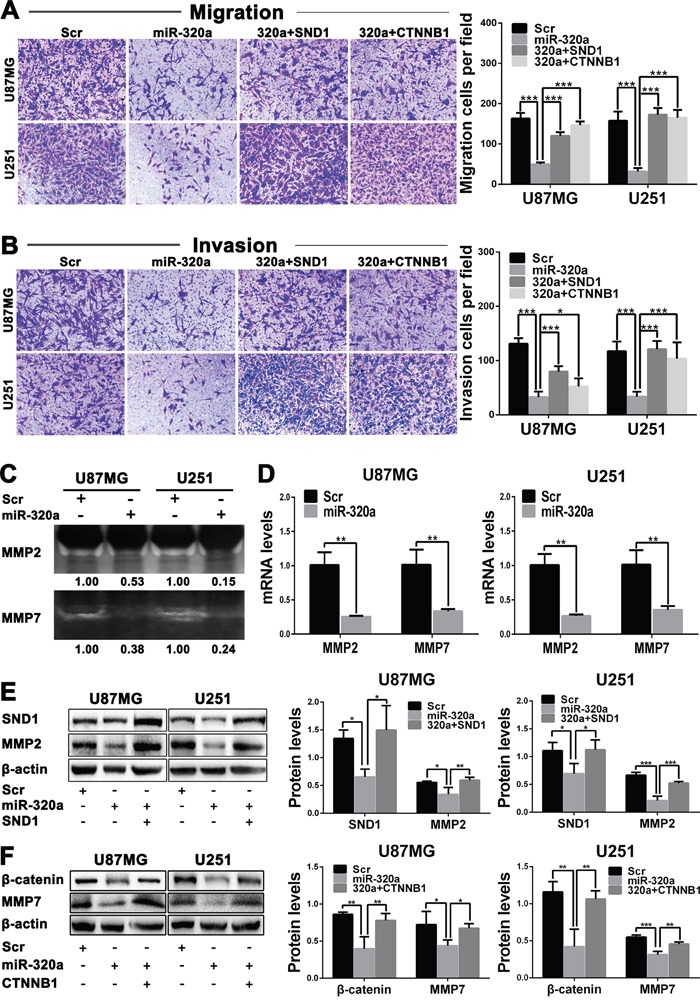
miR-320a inhibits the migration and invasion of GBM cells by targeting SND1 and β-catenin **A** and **B**. Representative images (left) and cell numbers (right) of migration(A) and invasion (B) of the indicated cells analyzed by transwell assay. **C**. MMP2 and MMP7 activities in culture medium of the indicated cells assessed by zymography. **D**. MMP2 and MMP7 mRNA levels in the indicated cells measured by qRT-PCR and normalized against GAPDH. The ratios of MMP2/GAPDH and MMP7/GAPDH in the Scr-transfected cells were arbitrarily set to 1.0. **E** and **F**. Protein levels of SND1, MMP2, β-catenin and MMP7 in the indicated cells detected by Western blot and normalized against β-actin. All the experiments were performed at least in triplicate and the data are presented as the mean ± SD. * *P*<0.05, ** *P*<0.01, *** *P*<0.001.

Transwell assays discovered that the migratory and invasive capacities of GBM cells were obviously weakened by miR-320a transfection (*P*<0.001) and partially recovered by SND1 and β-catenin overexpressions (*P*<0.05~0.001; Figure 5A and 5B). Zymography assay indicated that MMP2 and MMP7 activities were significantly lowered in the culture medium of miR-320a-transfected GBM cells (Figure 5C). Moreover, MMP2 and MMP7 mRNAs were also notably reduced in the miR-320a-transfected cells (*P*<0.01; Figure 5D). Western blot results verified that miR-320a transfection significantly decreased MMP2 and MMP7 proteins (*P*<0.05~0.001) through inhibiting SND1 or β-catenin expression (*P*<0.05~0.01) in GBM cells, and the suppressive effects of miR-320a could be reversed by SND1 or β-catenin overexpression (*P*<0.05~0.001; Figure 5E and 5F). These findings reveal that miR-320a reduces MMP2 and MMP7 by directly silencing SND1 or β-catenin, thereby suppressing the migration and invasion of GBM cells.

### miR-320a inhibits glioma invasion by weakening TGFβ1 pathway activity

**Figure 6:**
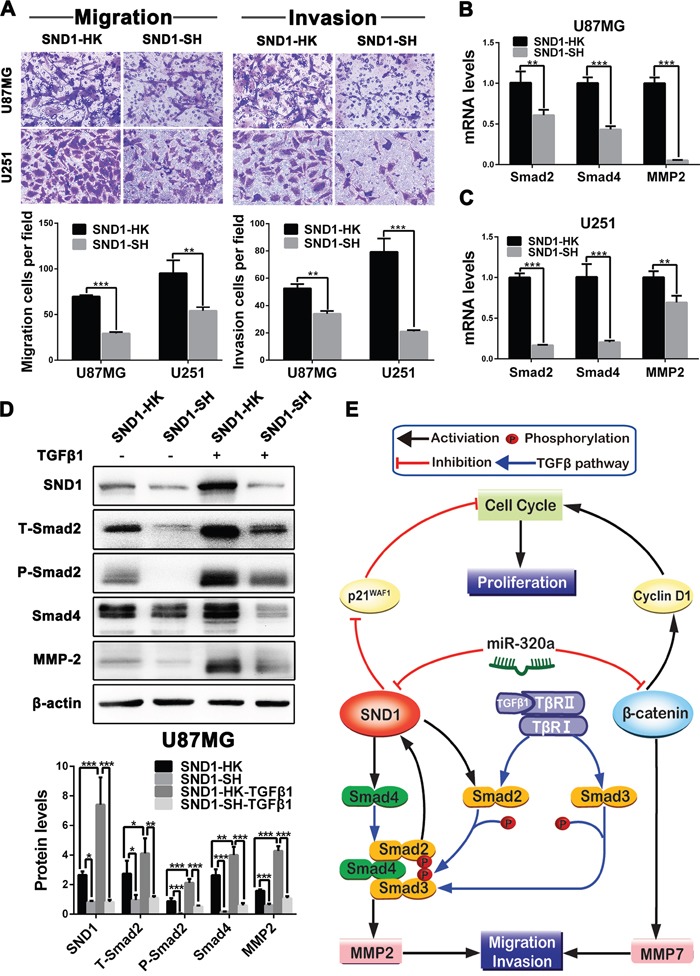
SND1 knockdown restrains the migration and invasion of GBM cells via weakening TGFβ1 pathway activity **A**. Representative images (upper) and numbers (under) of the indicated migratory and invasive cells analyzed by transwell assay. The SND1-knockdown (SND1-SH) and control (SND1-HK) sub-cell lines of U87MG and U251 cells were established by infecting lentiviruses to stably mimic the silencing effect of miR-320a. **B and C**. Smad2, Smad4 and MMP2 mRNA levels in the indicated cells detected by qRT-PCR and normalized against GAPDH. **D**. Protein levels of SND1, T-Smad2, P-Smad2, Smad4 and MMP2 in the indicated U87MG sub-cell lines untreated or stimulated with TGFβ1 measured by Western blot and normalized against β-actin. All the experiments were performed at least in triplicate and the data are presented as the mean ± SD. * *P*<0.05, ** *P*<0.01, *** *P*<0.001. **E**. Schematic illustration of the molecular pathways by which miR-320a suppresses the proliferation, migration and invasion of glioma cells.

To further clarify the underlying mechanisms of SND1 in miR-320a-induced MMP2 reduction and invasive suppression, we focused on Smad2 and Smad4, two pivotal downstream signaling proteins of SND1 in TGFβ1 pathway. We established the SND1-knockdown (SND1-SH) and control (SND1-HK) sub-cell lines of U87MG and U251 cells via infecting lentivirus expressing SND1 shRNA or scrambled control sequence, and validated the knockdown efficiencies by qRT-PCR and Western blot (Supplementary Figure 8A and 8B). In comparison with SND1-HK cells, SND1-knockdown significantly suppressed the migration and invasion of SND1-SH cells (*P*<0.01~0.001; Figure 6A), meanwhile decreased the mRNAs of Smad2, Smad4 and MMP2 in SND1-SH cells (*P*<0.01~0.001; Figure 6B and 6C). Moreover, SND1-knockdown obviously reduced the total Smad2 (T-Smad2), phosphorylated Smad2 (P-Smad2), Smad4 and MMP2 in SND1-SH cells (*P*<0.05~0.001; Figure 6D). Although TGFβ1 stimulus observably increased the SND1, T-Smad2, P-Smad2, Smad4 and MMP2 in SND1-HK cells (*P*<0.05~0.001), these effects of TGFβ1 were abrogated by SND1-knockdown in SND1-SH cells (*P*<0.01~0.001; Figure 6D). These results indicate that SND1 promotes MMP2 expression as a signaling amplifier of TGFβ1 pathway, and that miR-320a restrains the MMP2 overexpression induced by TGFβ1 pathway overactivation via directly silencing SND1, thereby inhibiting the migration and invasion of GBM cells (Figure 6E). Our current study reveals that miR-320a functions as an important suppressor for the cell proliferation, migration and invasion of malignant gliomas by directly silencing SND1 and β-catenin (Figure 6E).

## DISCUSSION

miR-320a may function as a tumor suppressor or promoter in different tumors [17, 18], but its exact roles and clinical relevance in gliomas remain indeterminate. In the present study, we identified miR-320a as a tumor suppressor to inhibit cell proliferation, migration and invasion in astrocytic gliomas. Mechanistically, we for the first time demonstrated that SND1 and β-catenin were direct functional targets of miR-320a in gliomas, which facilitated our understanding of the mechanisms underlying glioma malignant progression. Importantly, we found that miR-320a, SND1 and β-catenin, not only were correlated with one another, but also predicted the survival of glioma patients, highlighting their potential values as novel prognostic biomarkers in human gliomas.

The comprehensive analyses based on miRNAs and their regulatory networks have provided new ideas for searching clinical biomarkers correlating with glioma grades, specific histological and molecular subtypes or prognosis [19, 20]. Our present data verified that miR-320a expression was significantly decreased, while SND1 and β-catenin expressions were observably increased with the grade elevation in 120 human gliomas of WHO grade II-IV, and that the subgroups with higher miR-320a and lower SND1 and β-catenin had better prognoses in the glioma patients with the same grade, IDH status, age and KPS, suggesting that they were potential biomarkers in distinguishing glioma grades and specific biomarkers for prognostic-based glioma subclassification. The inverse correlation between miR-320a and SND1 or β-catenin implied that miR-320a downexpression resulted in SND1 and β-catenin overexpressions in gliomas. Furthermore, miRNAs are stable in FFPE samples and can be easily detected by ISH [21, 22]. Thus, miR-320a, SND1 and β-catenin could be the novel and clinical feasible candidates for glioma diagnosis and subclassification.

Malignant gliomas, of which 60 to 70% are GBM, are characterized by rapid growth and relentless invasion resulted from high-speed proliferation and migration of tumor cells [4, 22–24]. In our glioma specimens, the expression of Ki-67, a known proliferation marker, not only was significantly increased with glioma grade elevation, but also was inversely correlated with miR-320a expression and positively correlated with SND1 and β-catenin expressions. Our *in vitro* results showed that miR-320a could effectively suppress the proliferation, migration and invasion of GBM cells. These facts indicated that miR-320a was a glioma suppressor, and suggested that SND1 and β-catenin overexpressions induced by miR-320a downexpression were important causes leading to the unlimited proliferation, migration and invasion of malignant glioma cells, highlighting the potential values of miR-320a, SND1 and β-catenin in the therapy of malignant gliomas.

β-catenin is an important glioma promoter [25], while recent studies have discovered that SND1 promotes oncogenesis and progression through increasing TGFβ1 pathway activity [26–28]. In Wnt signal-on state, β-catenin translocates to the nucleus and facilitates cyclin D1 and MMP7 expressions by forming a complex with other transcriptional activators [29–31]. SND1 as a transcriptional coactivator stimulates the expressions of Smad2 and Smad4 in TGFβ1 pathway [28]. P-Smad2 and P-Smad3 phosphorylated by TGFβ1 signaling form a complex with Smad4 and facilitate SND1 and MMP2 expressions via activating their gene transcriptions [27, 32, 33]. Additionally, SND1 also suppresses p21^WAF1^ expression through enhancing the activity of RNA-induced silencing complex [34]. The cyclin D1 increase and p21^WAF1^ decrease accelerate cell G1/S phase transition and proliferation, whilst MMP2 and MMP7 overexpressions expedite cell migration and invasion by degrading extracellular matrix.

We identified SND1 and β-catenin as direct functional targets of miR-320a by the analysis of TCGA data, bioinformatics prediction, luciferase reporter assay, qRT-PCR and Western blot. Subsequently, we confirmed that miR-320a-induced knockdowns of SND1 and β-catenin significantly increased p21^WAF1^ or decreased cyclin D1, and reduced the expressions and extracellular activities of MMP2 and MMP7 of GBM cells, consequently suppressing their G1/S phase transition, proliferation, migration and invasion. All these findings were further validated by the rescue experiments. Furthermore, we proved that shRNA knockdown of SND1 not only reduced the T-Smad2, P-Smad2, Smad4 and MMP2 in GBM cells, but also eliminated the positive regulatory effects of TGFβ1 on these proteins and SND1. Meanwhile, shRNA knockdown of SND1 perfectly imitated the suppressive effects of miR-320a on migration and invasion of GBM cells by decreasing Smad2, Smad4 and MMP2 mRNAs. Combining the inverse relevance between miR-320a and SND1 or β-catenin in the glioma specimens, our results indicated that SND1 and β-catenin overexpressions induced by miR-320a downexpression could decrease p21^WAF1^ and also increase MMP2, MMP7 and cyclin D1 by enhancing the activities of TGFβ1/Smad and Wnt/β-catenin pathways, thereby accelerating the cell proliferation and invasion of malignant gliomas (Figure 6E).

Our study indicated that miR-320a was decreased in gliomas, especially in GBM. Recent studies have shown that miR-320a promoter may directly bind with transcriptional regulation factor ETS-1 and long noncoding RNA NLC1-C, and may also be methylated, which both repress miR-320a transcription in cancer cells [35, 36]. However, the molecular mechanism of miR-320a downexpression remains unknown in gliomas. Further studies are underway to investigate the unknown mechanism. Our multivariate analysis showed that β-catenin was not an independent predictor for DFS and OS of glioma patients, but univariate analysis demonstrated that β-catenin was an auxiliary predictor of the patients’ survival. Since miR-320a may also exert anti-glioma effects by silencing other targets [37, 38] and SND1 is a pivotal multifunctional protein promoting oncogenesis and progression [26], the prognostic significance of β-catenin is not as important as those of miR-320a and SND1 in gliomas.

In summary, our study revealed that miR-320a inhibited the proliferation and invasion of glioma cells by directly targeting SND1 and β-catenin, and predicted better prognosis in human gliomas, especially in GBM. More importantly, miR-320a might be a novel biomarker for molecular subclassification of malignant gliomas and a therapeutic candidate for these lethal diseases.

## MATERIALS AND METHODS

### Tissue samples and clinicopathological data

The surgical specimens of 120 astrocytic gliomas and 20 nontumoral brain tissues (control) were collected from Tianjin Medical University General Hospital (TMUGH) with written consent. After surgical excision, specimens were fixed in 3.7% buffered formaldehyde solution immediately and embedded in paraffin afterwards (FFPE samples). Then, 5μm continuous sections were prepared for HE staining, miR-320a *in situ* hybridization and the immunohistochemical detections of SND1, β-catenin and Ki-67. Histopathological diagnoses were independently made by two neuropathologists according to the 2016 World Health Organization (WHO) classification of central nervous system tumors [39]. The gene mutations of isocitrate dehydrogenase 1 and 2 (IDH1/2) were detected using Sanger sequencing to ascertain the genetic types of our gliomas (Supplementary Figure 9). The WHO grades, IDH1/2 status and patients’ clinical features were summarized in Supplementary Table 3. All the 120 glioma patients had complete information and were followed up after operation until December 31, 2013, with a follow-up time of 4.5 to 89 months. This study was carried out in accordance with the principles of the Helsinki Declaration and approved by the Ethics Committee of TMUGH.

An independent cohort of 391 patient specimens from The Cancer Genome Atlas (TCGA) database (https://cancergenome.nih.gov/) was used to validate the correlation between miR-320a expression and DFS or OS in GBM.

### *In situ* hybridization (ISH) and immunohistochemistry (IHC)

For ISH detection, deparaffinized tissue sections were hybridized with 50 nM LNA-modified and digoxin-labeled miR-320a oligonucleotide probe (Exiqon, Vedbaek, Denmark; Supplementary Table 4) for 1 h at 55°C, incubated with 5μg/ml anti-digoxin- Rhodamine antibody (Roche Applied Science, Indianapolis, IN, USA) overnight at 4°C, and stained with DAPI in the dark for 15 min at room temperature (RT). The IHC staining was performed according to the standard ABC protocol with the antibodies of mouse anti-human SND1 (Abcam, Cambridge, MA, USA), rabbit anti-human β-catenin (CST, Boston, MA, USA) and rabbit anti-human Ki-67 (Millipore, Billerica, MA, USA). The labeling index (LI) is presented as the percentage of positive cell number to total cell number.

### Cell culture, lentivirus and stable-infected cell line establishment

Human GBM cell lines, U118 and U87MG cells were obtained from the American Type Culture Collection (ATCC), and LN308, U251 and SNB19 cells were purchased from the China Academia Sinica Cell Repository. The GBM cell lines (TJ905 and TJ899) from Chinese patients were established and maintained by our lab. The immortalized human astrocyte cell line (UC2) was used as a nontumoral control. All the cells were cultured in Dulbecco's Modified Eagle Medium (DMEM; Gibco, Grand Island, NY, USA) containing 10% FBS (Gibco) at 37°C in a humidified incubator with 5% CO_2_.

The recombinant lentiviruses expressing shRNA targeting SND1 (SND1-SH) or scrambled control sequence (SND1-HK; Supplementary Table 4) were constructed and packaged by GenePharma (Shanghai, China). U87MG and U251 sub-cell lines were established by infecting the cells with two lentiviruses. For TGFβ1 stimulation, the U87MG sub-cell lines were incubated with TGFβ1 (10 ng/ml) for 24 h.

### Oligonucleotides, plasmids and transient transfection

miR-320a mimics (miR-320a) and a corresponding scrambled control sequence (Scr; Supplementary Table 5) were synthesized by Ribobio (Guangzhou, China). The plasmids expressing SND1 (pSG5-SND1) and CTNNB1 (pCI-neo-β-catenin WT) were constructed as previously described and verified by DNA sequencing [40, 41]. The U87MG and U251 cells of miR-320a group (miR-320a) and control group (Scr) were transfected with the corresponding dsRNA oligonucleotides using X-tremeGENE siRNA Transfection Reagent (Roche) at a final concentration of 50 nM. The cells of rescue groups were transfected with miR-320a plus pSG5-SND1 (320a+SND1) or pCI-neo-β-catenin WT (320a+CTNNB1).

### Wound healing and transwell assays

Uniform artificial wounds were made 2 d after transfection, and the cells were cultured for another 48 h. Cell migration ability is represented by the wound gap distance in microscopic field (×40) at the time points of 0, 12, 24, and 36 h. For transwell assays, 24 h after transfection, the cells (3×10^4^/well) were seeded into the upper well of the transwell chamber coated with or without Matrigel (BD Bioscience, Mountain View, CA, USA) and allowed to migrate or invade towards the medium containing 10% FBS for 24 h. The cells that reached the lower surface were fixed with methanol and stained with 0.1% crystal violet. The cells were counted in 9 randomly selected microscopic fields (×400) from each chamber.

### Cell proliferation assays

U87MG and U251 cells (1.5×10^3^/well) were seeded into 96-well plates after transfection and incubated for another 5 or 6 d. At each 24 h interval, Cell Counting Reagent-8 (10 μl/well; Beyotime, Suzhou, China) was added and the absorbance at 450 nm was measured with a Synergy microplate reader (BioTek Instruments, Winooski, VT, USA). For colony formation assay, U251 cells (1×10^3^/well) were seeded 2 d after transfection and incubated for 14 d. The colony formation efficiency was calculated as colony number / inoculated cell number × 100%. For 5-ethynyl-2′-deoxyuridine (EdU) assay, 50 mM of the reagent (Cell Light EdU DNA imaging Kit; RiboBio) was added 2 d after transfection, and the cells were stained according to the manufacturer's instructions. The EdU-positive rate was calculated as EdU positive cells / Hoechst-stained cells × 100%.

### Flow cytometry assay (FCM)

Two days after transfection, the cells were harvested and fixed with 70% ethanol at 4°C overnight. The cells were incubated with propidium iodide (40 μg / ml; Sigma, St. Louis, MO, USA) for 30 min and analyzed with a FACSCalibur™ Flow Cytometer (Becton Dickinson, Franklin Lakes, NJ, USA). The data were processed with the ModFit LT software (Verity Software House, Topsham, ME, USA).

### Bioinformatic prediction and target selection

The genes of co-expression and highly inverse correlation with miR-320a in GBM were optimized as the candidates of miR-320a targets by analyzing the expression profile data from TCGA database. Targetscan (http://www.targetscan.org/), miRTarBase (http://mirtarbase.mbc.nctu.edu.tw/) and PicTar (http://pictar.mdc-berlin.de/cgi-bin/PicTar_vertebrate.cgi) were used to further verify whether the candidates were the potential targets of miR-320a. Subsequently, the functions of the potential targets in gliomagenesis were assessed by reference review and the important ones were identified as the crucial targets of miR-320a in gliomas.

### Dual-luciferase reporter assay

The cDNAs coding SND1 or β-catenin 3′-untranslated region (3′-UTR; p-WT) and their mutants (p-MT) without the putative miR-320a binding sites were inserted downstream of the firefly luciferase reporter gene in the pEZX-MT01 vectors (GeneCopoeia, Guangzhou, China). The recombinant plasmids were used to transfect U87MG or U251 cells alone (Mock) or with the miR-320a and Scr using X-tremeGENE HP DNA Transfection Reagent (Roche). The activities of firefly and renilla luciferases were measured with the Dual-luciferase Reporter Assay System (Promega, Fitchburg, WI, USA). The results were presented as the firefly luciferase activities normalized against those of renilla.

### Quantitative RT-PCR (qRT-PCR)

qRT-PCR detection was performed as previously described [24]. miR-320a levels in UC2 cell line and 7 human GBM cell lines (LN308, U118, U251, SNB19, TJ905, TJ899, U87MG) were quantified by stem-loop qRT-PCR with U6 as the internal control (Ribobio). The GoTaq qPCR Master Mix Kit (Promega) was used to quantify the mRNAs involved in this study and GAPDH was used as the internal control. The sequences of the specific primers are listed in Supplementary Table 6. The fold changes of miRNA and mRNA levels were calculated by the 2^−ΔΔCt^ method.

### Western blotting

Western blotting was carried out as previously described [24]. Rabbit anti-human β-catenin, MMP7, cyclin D1, Smad2 and phosphorylated Smad2 (CST), mouse anti-human SND1 (Abcam), Smad4 (R&D Systems, Minneapolis, MN, USA), MMP2, p21^WAF1^ and β-actin (Boster Biological Technology, Wuhan, China) were used as primary antibodies.

### Metalloproteinase zymography

The conditioned media collected from each group were concentrated and separated in 10% or 12% SDS-polyacrylamide gels containing 1 mg/ml of gelatin or β-casein (Sigma, USA) under non-reducing condition. Then, the gels were rinsed in renaturing buffer, incubated with developing buffer and stained with 0.5% Coomassie blue R-250. The presences of MMP2 and MMP7 were indicated as clear and unstained bands against the dark background.

### Statistical analysis

Statistical analyses were performed using SPSS 21.0 software (IBM, Chicago, Illinois, USA). One-way ANOVA test, Student *t* test, Pearson correlation analysis, Kaplan-Meier analysis, log-rank test and Cox's proportional hazards regression model were used to analyze corresponding data in this study. Results were presented as the mean ± standard deviation (SD). Statistical significance was assigned at *P*<0.05 (*/^25B2^), *P*<0.01 (**/^25B225B2^) or *P*<0.001 (***/^25B225B225B2^). All the experiments of cell lines were performed at least three times with triplicate samples.

## SUPPLEMENTARY MATERIALS FIGURES AND TABLES


