# Supplementary material for: miR-320a functions as a suppressor for gliomas by targeting SND1 and β-catenin, and predicts the prognosis of patients
Source: Oncotarget. 2017 Feb 1;8(12):19723–37. doi: 10.18632/oncotarget.14975 (PMC5386717; doi:10.18632/oncotarget.14975)
Supplement: Supplementary file 1 [file oncotarget-08-19723-s001.pdf]

miR-320a functions as a suppressor for gliomas by targeting SND1 and β-catenin, and predicts the prognosis of patients

SUPPLEMENTARY FIGURES AND TABLES

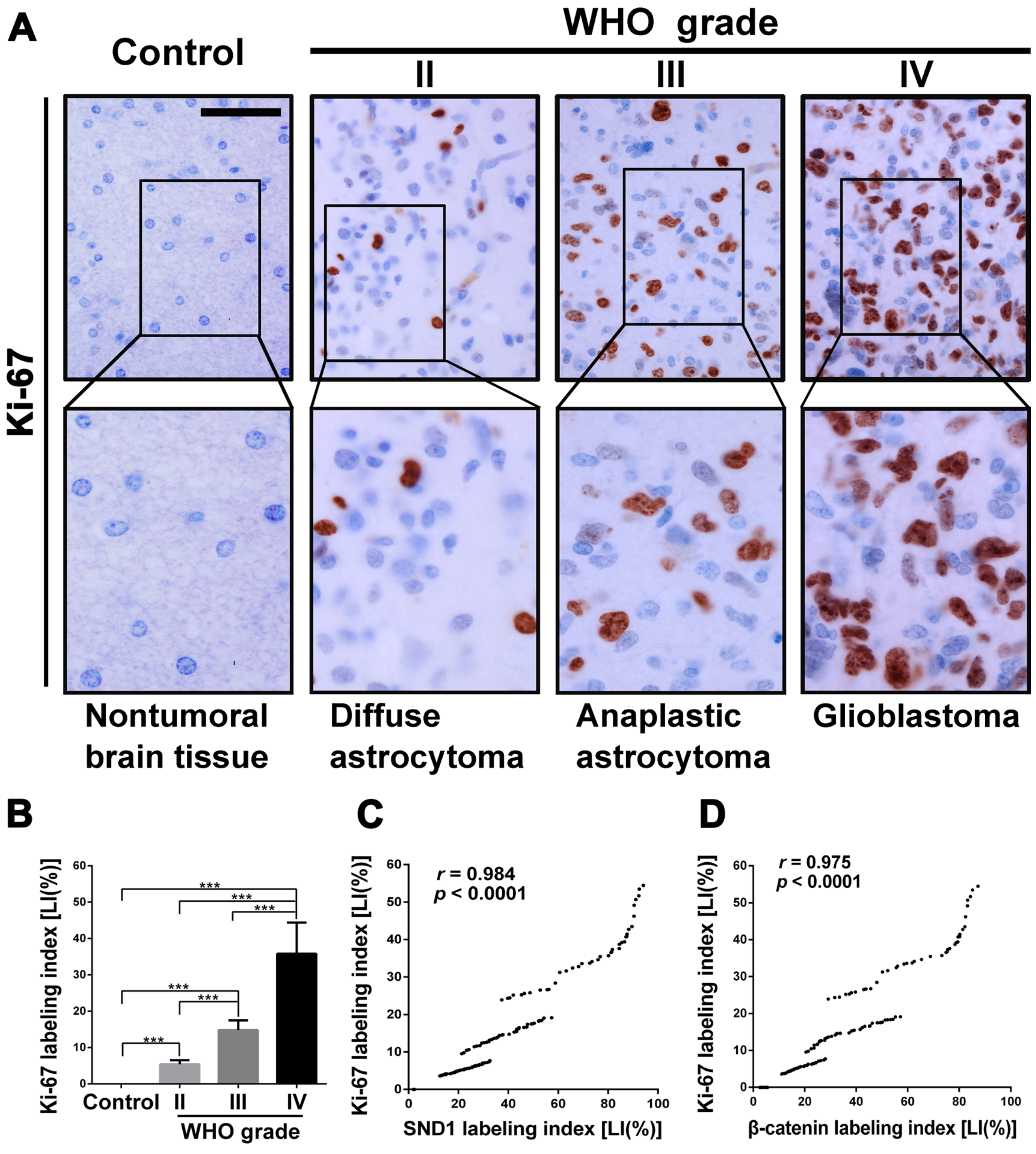

Supplementary Figure 1: Ki-67 expression correlates with glioma grades and the expressions of SND1 and β-catenin. **A.** Representative images of Ki-67 IHC detection. Scale bar, 50 μm. **B.** Comparisons among groups of Ki-67 expression level [Labeling index (%), LI] in the FFPE samples of 120 gliomas and 20 nontumoral control brain tissues. The Ki-67 LI of each sample was calculated with Leica Image Pro Plus 5.0 software according to the percentage ratio of positive cell number to total cell number and the data in (B) are presented as the mean ± SD; *** P<0.001. **C** and **D.** Pearson correlation analysis between Ki-67 LIs and SND1 LIs (C) or β-catenin LIs (D) in the FFPE samples as indicated.

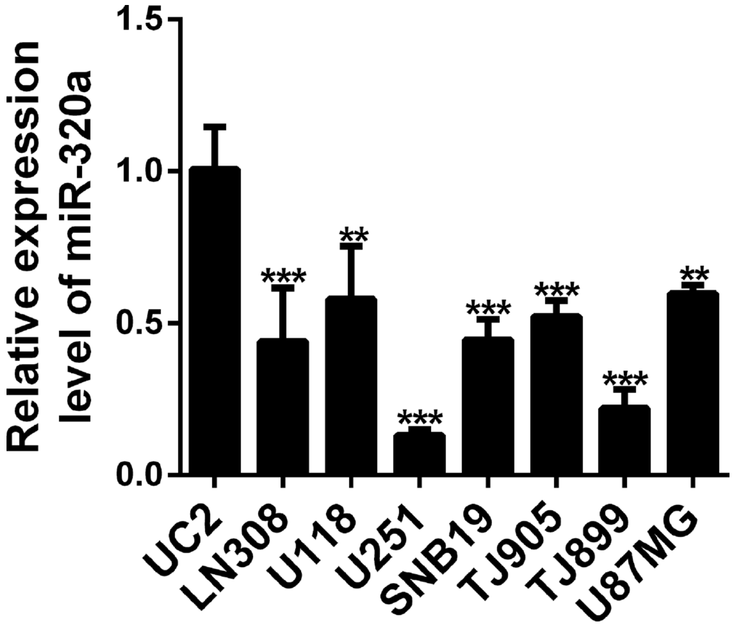

Supplementary Figure 2: miR-320a expression level in UC2 and seven GBM cell lines. miR-320a levels in the indicated cell lines quantified by stem loop qRT-PCR and normalized against U6. The miR-320a/U6 ratio in immortalized human astrocyte cell line UC2 was arbitrarily set to 1.0. The detection of each cell line was repeated at least three times and the data are presented as the mean ± SD; ** $P<0.01$, *** $P<0.001$.

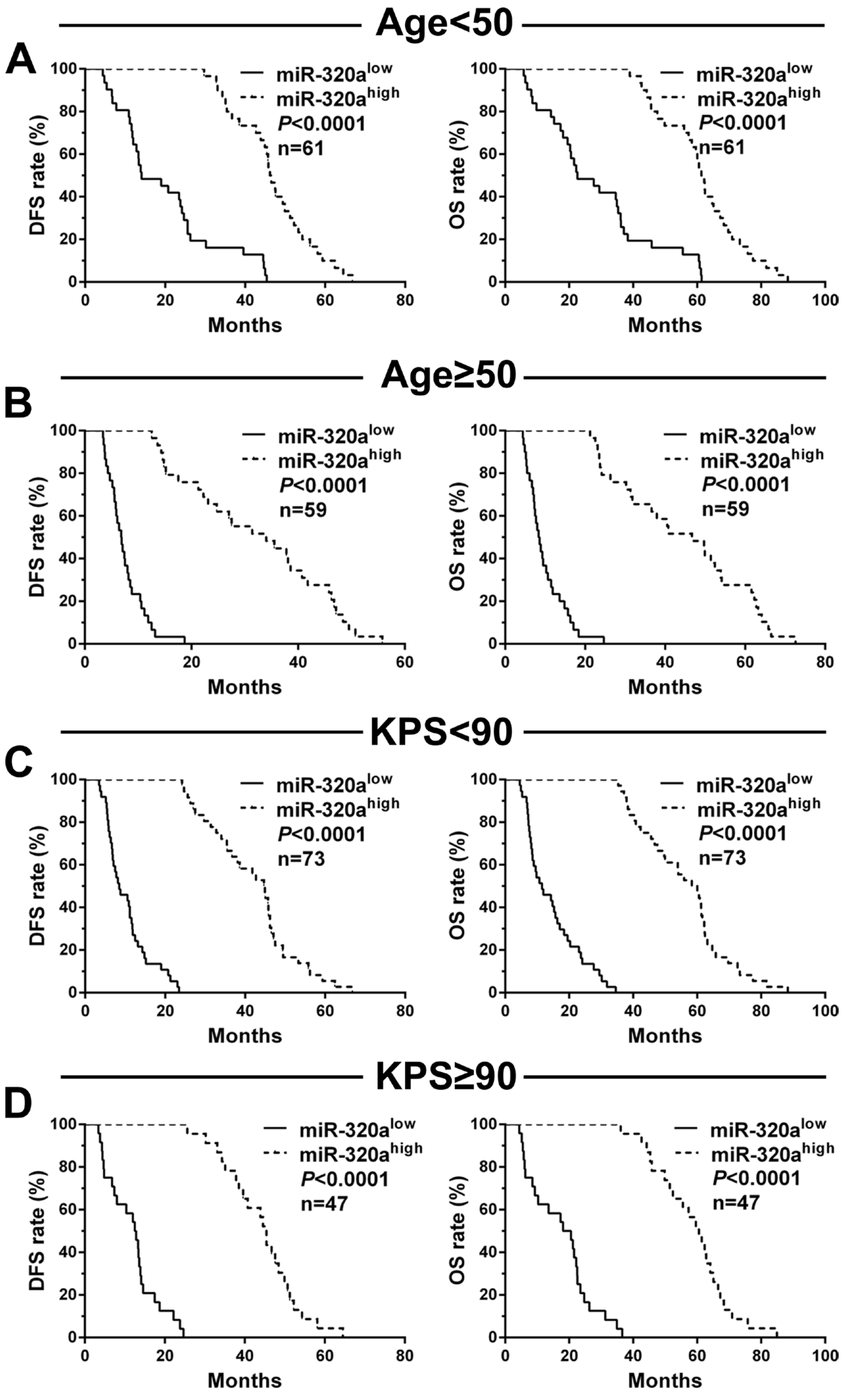

Supplementary Figure 3: miR-320a expression correlates with the prognoses of glioma patients in the same age and KPS groups. A-D. Kaplan-Meier analysis of the correlation between miR-320a and DFS (left) or OS (right) of the glioma patients in age < 50 (A), age ≥ 50 (B), KPS < 90 (C) and KPS ≥ 90 (D) groups. Patients were stratified into high and low expression subgroups using the median of miR-320a LIs.

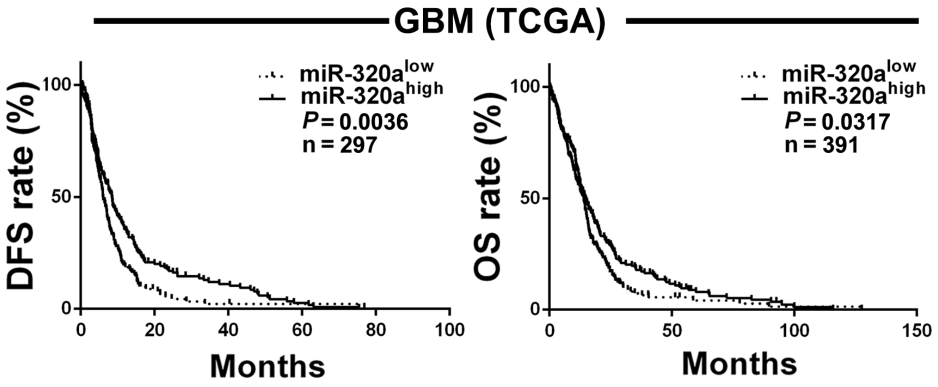

Supplementary Figure 4: miR-320a expression correlates with the prognosis of GBM patients in TCGA database. Kaplan-Meier analysis of the correlation between miR-320a and DFS (left) or OS (right) of the GBM patients. Patients were stratified into high and low expression subgroups using the median of relative miR-320a levels.

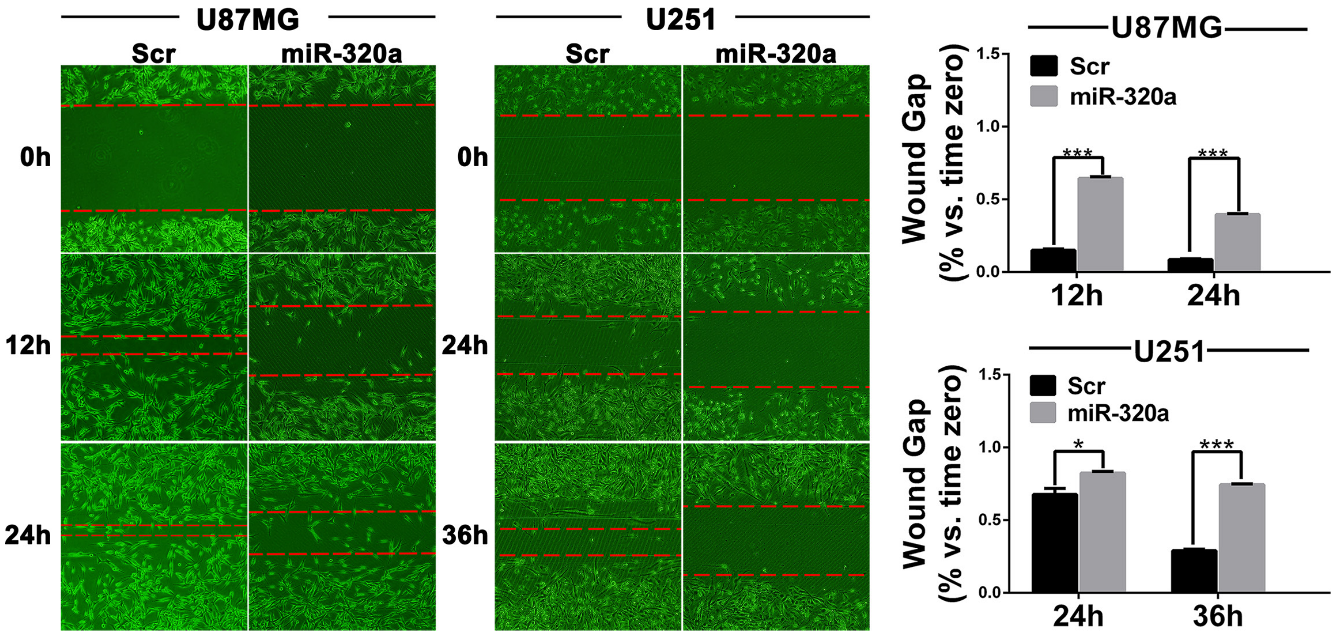

Supplementary Figure 5: miR-320a reduces the migratory capacity of GBM cells. Representative images (left and middle) and comparisons among groups (right) of migratory capacities of U87MG and U251 cells transfected with scrambled control sequence (Scr) or miR-320a mimics (miR-320a) evaluated by wound healing assay. The wound edges were outlined by horizontal lines and the migratory capacities of the indicated cells were quantified according to (wound width at each indicated time point/wound width at 0 hour) ×100%. All the experiments were performed at least in triplicate and the data are presented as the mean ± SD; * P<0.05, *** P<0.001.

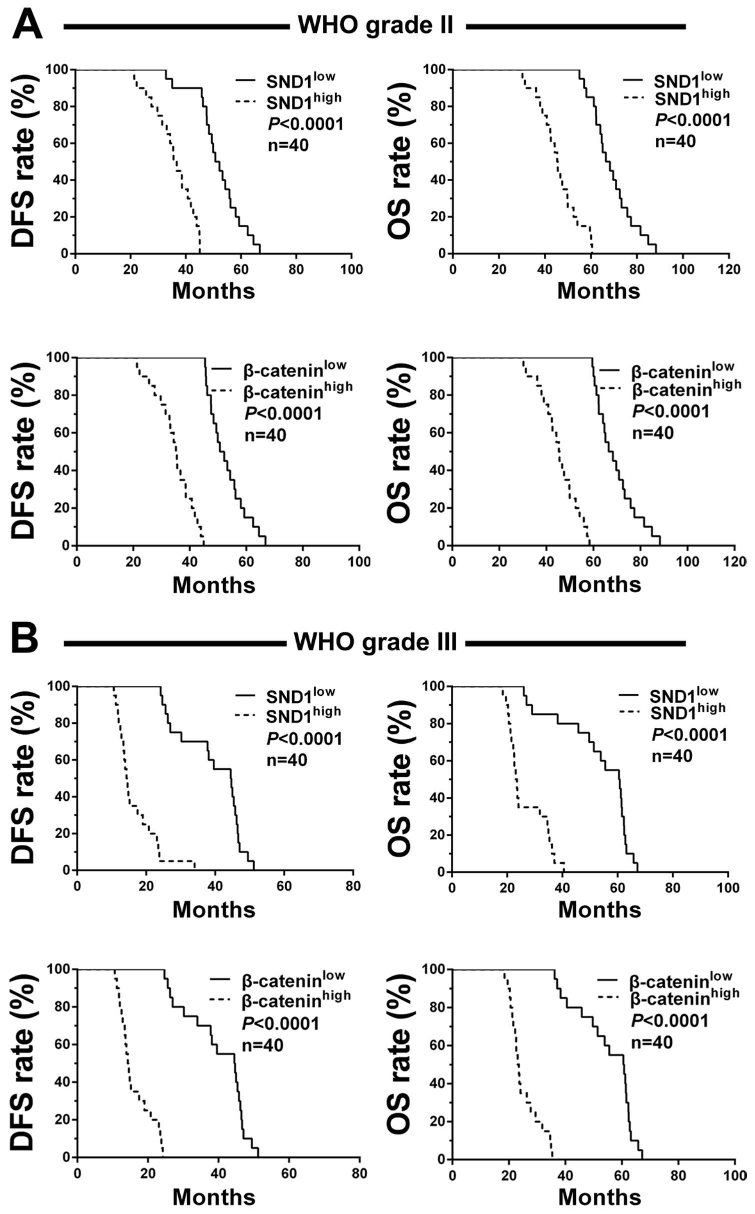

Supplementary Figure 6: SND1 and β-catenin expressions correlate with the prognoses of patients with grade II and III gliomas. A and **B.** Kaplan-Meier analysis of the correlation between SND1 or β-catenin and DFS (left) or OS (right) of the patients with WHO grade II (A) and grade III (B) gliomas. Patients were stratified into high and low expression subgroups using the median of SND1 or β-catenin LIs.

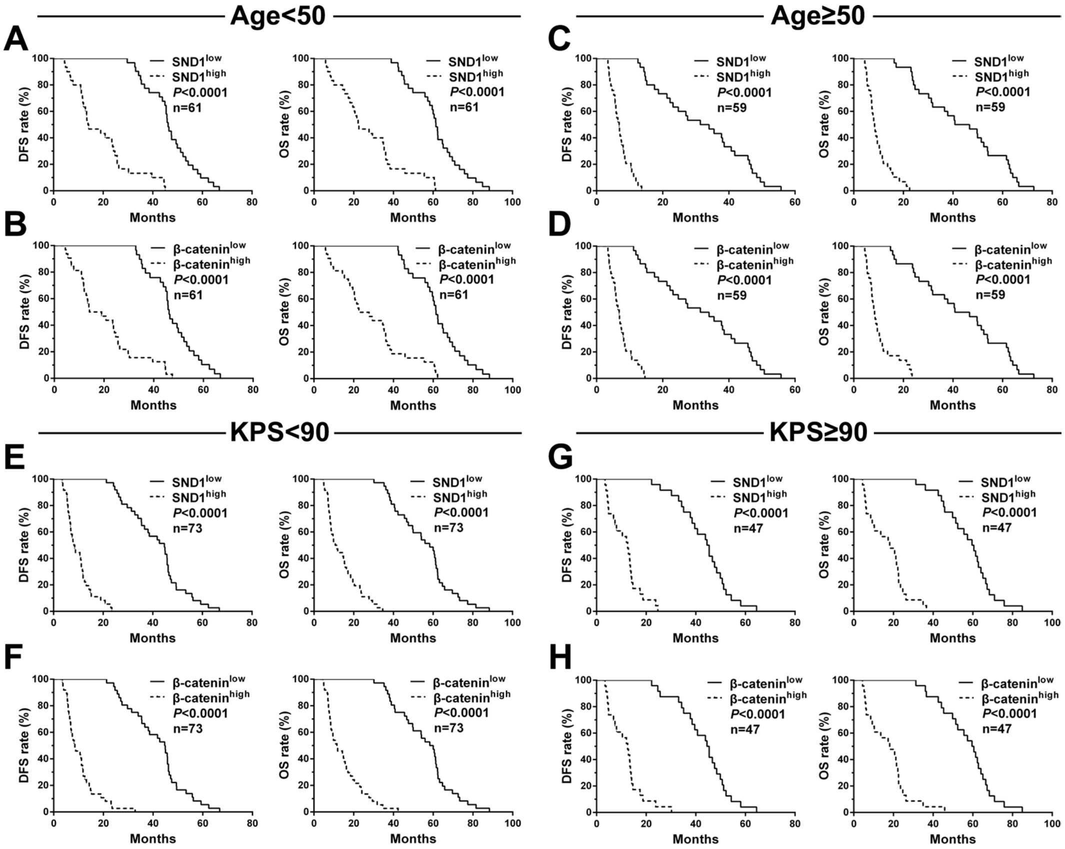

Supplementary Figure 7: SND1 and β-catenin expressions correlate with the prognoses of glioma patients in the same age and KPS groups. A-D. Kaplan-Meier analysis of the correlation between SND1 or β-catenin and DFS (left) or OS (right) of the glioma patients in age < 50 (A and B) and age ≥ 50 (C and D) groups. **E-H.** Kaplan-Meier analysis of the correlation between SND1 or β-catenin and DFS (left) or OS (right) of the glioma patients in KPS < 90 (E and F) and KPS ≥ 90 (G and H) groups. Patients were stratified into high and low expression subgroups using the median of SND1 or β-catenin LIs.

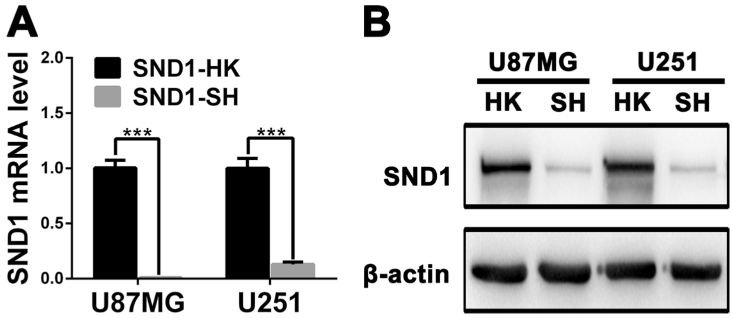

Supplementary Figure 8: SND1 knockdown efficiency in the GBM sub-cell lines infected with lentiviruses. A. SND1 mRNA levels in U87MG and U251 sub-cell lines infected with lentiviruses expressing SND1 shRNA (SND1-SH) or scrambled control sequence (SND1-HK) measured by qRT-PCR and normalized against GAPDH. The ratio of SND1/GAPDH in SND1-HK cells was arbitrarily set to 1.0. **B.** SND1 protein levels in the indicated sub-cell lines detected by Western blot and normalized against β-actin. All the experiments were performed at least in triplicate and the data in (A) are presented as the mean ± SD; $***P < 0.001$.

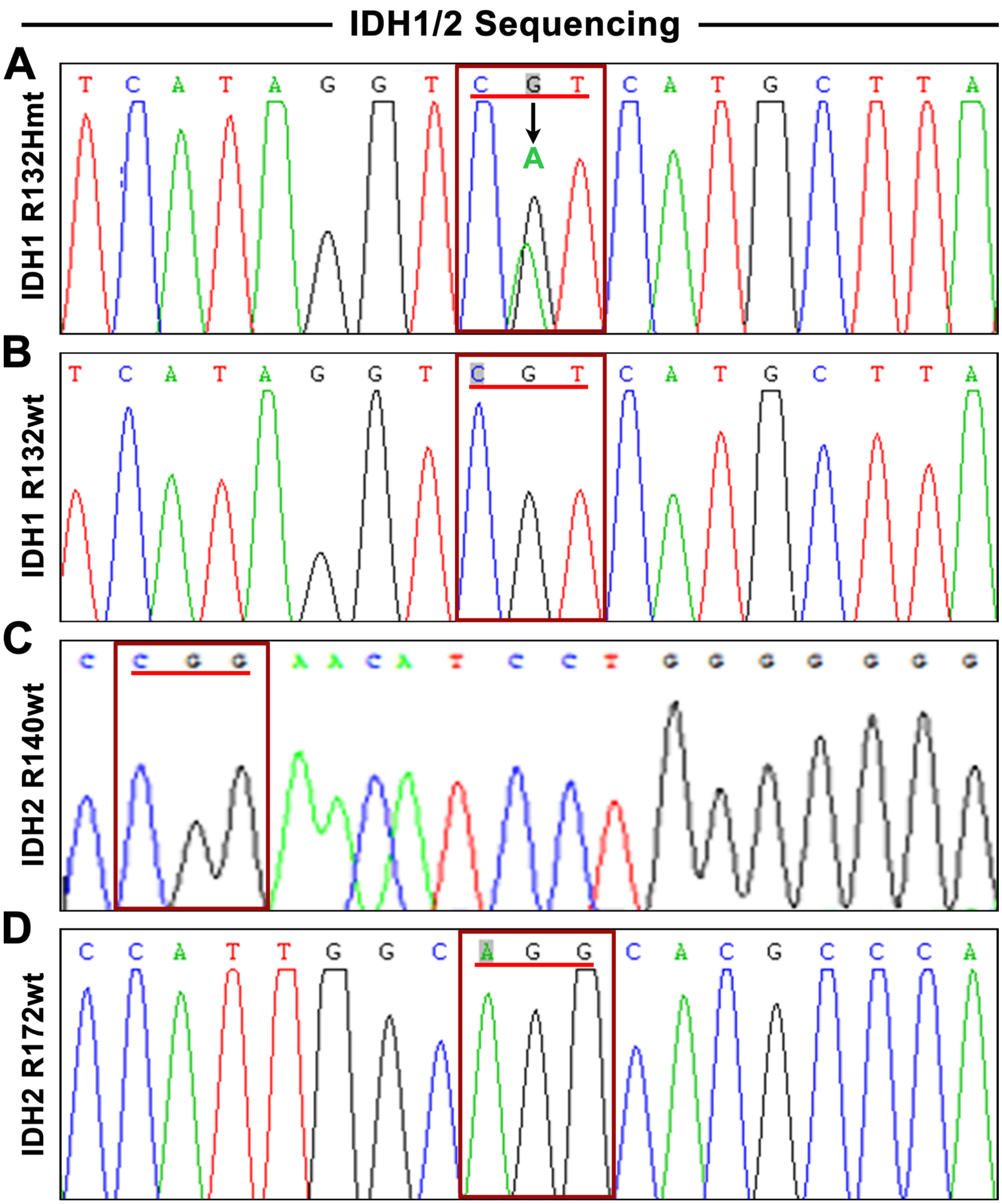

Supplementary Figure 9: Sanger sequencing of *IDH1* and *IDH2* genes in glioma tissue specimens. A-D. Representative examples of IDH1 R132H mutation (A), IDH1 R132 wild type (B), IDH2 R140 wild type (C) and IDH2 R172 wild type (D).

Supplementary Table 1: Univariate analysis for DFS and OS in patients with gliomas

Factors	DFS		OS	
	HR(95%CI)	*P*	HR(95%CI)	*P*
Gender	1.087 (0.752-1.571)	0.656	1.081(0.748-1.562)	0.677
Age	1.022 (1.008-1.036)	0.002	1.021(1.007-1.035)	0.003
Predominant side	0.963 (0.713-1.301)	0.806	0.953(0.705-1.289)	0.756
Predominant location	1.291 (1.022-1.630)	0.032	1.283(1.016-1.620)	0.037
KPS	0.994 (0.972-1.016)	0.585	0.994 (0.972-1.016)	0.582
IDH status	0.051 (0.028-0.093)	<0.0001	0.042(0.023-0.077)	<0.0001
miR-320a LI	0.686 (0.640-0.735)	<0.0001	0.699(0.654-0.748)	<0.0001
SND1 LI	1.507 (1.406-1.616)	<0.0001	1.389 (1.314-1.469)	<0.0001
β-catenin LI	1.336 (1.271-1.405)	<0.0001	1.282(1.227-1.339)	<0.0001
Ki-67 LI	1.345 (1.284-1.408)	<0.0001	1.348(1.287-1.411)	<0.0001

Abbreviations: HR, hazard ratio; CI, confidence interval; KPS, Karnofsky performance score; LI, labeling index.

Supplementary Table 2: Correlation analysis between expressions of miR-320a and predicted potential targets in GBM

No.	Gene Symbol	Pearson Score	*P* value
1.	KITLG	−0.41	4.25e-3
2.	SND1	−0.36	5.31e-3
3.	CTSV	−0.29	7.46e-3
4.	STARD4	−0.26	0.00204
5.	CTNNB1	−0.26	0.00331
6.	SYNGR2	−0.26	0.00971
7.	CD3G	−0.24	0.0107
8.	CGA	−0.23	0.0346
9.	ITGB1	−0.23	0.0363
10.	HECTD2	−0.22	0.0299
11.	RBP7	−0.21	0.0287
12.	CUTC	−0.20	0.0365
13.	TFRC	−0.20	0.0483
14.	RGS10	−0.16	0.0468
15.	APEX1	−0.13	0.0526
16.	ETFA	−0.12	0.0860
17.	RBM24	−0.12	0.131
18.	ZFHX3	−0.12	0.0865
19.	MBIP	−0.11	0.263
20.	POLR1C	−0.11	0.373
21.	YWHAH	−0.11	0.0841
22.	ARFIP1	−0.10	0.248
23.	FBXO28	−0.09	0.693
24.	SMIM19	−0.09	0.870
25.	C1orf145	−0.08	0.742
26.	KCNS3	−0.08	0.715
27.	MED7	−0.04	0.457
28.	MTRF1	−0.04	0.684
29.	DNAI1	−0.03	0.696
30.	CDCA3	−0.02	0.378
31.	DPY30	−0.02	0.243
32.	ZNF214	0.03	0.893
33.	ZWILCH	0.03	0.856
34.	DAZAP1	0.04	0.670
35.	DNAH7	0.04	0.757
36.	PCGF1	0.04	0.779
37.	RPA3-AS1	0.04	0.551
38.	RCN2	0.06	0.317
39.	FAM89A	0.07	0.306
40.	MPPED2	0.09	0.420
41.	PBX3	0.09	0.829
42.	ASAH2C	0.10	0.374
43.	GCG	0.11	0.641
44.	TMEM98	0.11	0.706
45.	ZNF23	0.14	0.658
46.	KRTAP4-1	0.24	0.381
47.	C6orf118	0.27	0.453

Notes: The data from expression profile chips of miRNA and mRNA of 607 GBMs in TCGA database

Supplementary Table 3: The clinicopathological characteristics of the glioma patients enrolled in this study

Feature	WHO Grade		
	II (n=40)	III (n=40)	IV (n=40)
IDH status			
Mutant type (IDH1 R132H)	35	33	2
Wild type (IDH1/2)	5	7	38
Gender			
Male	22	22	27
Female	18	18	13
Age (Year, Mean±SD)	43±11.9	48±15.2	56±13.0
Age <50	29	22	10
Age ≥50	11	18	30
Predominant side			
Left	20	17	20
Right	17	21	18
Middle	3	2	2
Predominant location			
Frontal lobe	30	26	19
Temporal lobe	6	8	13
Parietal lobe	1	3	5
Occipital lobe	1	1	1
Cerebellum	2	1	1
CPA	0	0	1
Third ventricle	0	1	0
KPS Score			
<90	23	24	26
≥90	17	16	14

Abbreviation: SD, Standard deviation.

Supplementary Table 4: The probe used for ISH and the inserted sequences of the recombinant lentiviruses

Oligonucleotides	Sequence
Digoxin-labeled LNA miR-320a probe	5'-TCGCCCTCTCAACCCAGCTTTT-3'
SND1-SH, antisense sequence	5′- GGTACCATCCTTCATCCAAAT -3′
SND1-HK, scramble control sequence	5′- TTCTCCGAACGTGTCACGT -3′

Supplementary Table 5: miR-320a mimics and scrambled control sequence

dsRNA	Sequence
miR-320a mimics	5′- AAAAGCUGGGUUGAGAGGGCGA -3′
	3′- UUUUCGACCCAACUCUCCCGCU -5′
Scrambled control sequence	5′-UUCUCCGAACGUGUCACGUTT-3′
	3′-TTAAGAGGCUUGCACAGUGCA-5′

Supplementary Table 6. Primers used for mRNA qRT-PCR detection

Primers	Orientation	Sequence
SND1	forward	5′- CTTATCACCTTCTTGCTTGCAG -3′
	reverse	5′- AAAGTGTAGCTTCCTCGCTGA -3′
β-catenin	forward	5′- GATTTGATGGAGTTGGACATGG -3′
	reverse	5′- TGTTCTTGAGTGAAGGACTGAG -3′
MMP2	forward	5′- CCAACTACAACTTCTTCCCTCGC -3′
	reverse	5′- AGCAAAGGCATCATCCACTGTC -3′
MMP7	forward	5′- AAACTCCCGCGT CAT AGA AAT -3′
	reverse	5′- TCCCTAGACTGCTACCATCCG -3′
SMAD2	forward	5′- AACAGAACTTCCGCCTCTGG -3′
	reverse	5′- GGAGGTGGCGTTTCTGGAAT -3′
SMAD4	forward	5′- ACGAACGAGTTGTATCACCTGG -3′
	reverse	5′- ATGGCTGTCCCTCAAAGTCAT -3′
GAPDH	forward	5′-TGCACCACCAACTGCTTAGC-3′
	reverse	5′-GGCATGGACTGTGGTCATGAG-3′
